# Clinical inertia in general practice, a matter of debate: a qualitative study with 114 general practitioners in Belgium

**DOI:** 10.1186/s12875-015-0221-1

**Published:** 2015-02-06

**Authors:** Isabelle Aujoulat, Patricia Jacquemin, Michel P Hermans, Ernst Rietzschel, André Scheen, Patrick Tréfois, Elisabeth Darras, Johan Wens

**Affiliations:** Université catholique de Louvain, Institute of Health & Society, Brussels, Belgium; Université Catholique de Louvain, Institute of Experimental and Clinical Research, Cliniques universitaires Saint-Luc, Department of Endocrinology and Nutrition, Brussels, Belgium; Ghent University, Department of Cardiovascular Diseases, and Department of Public Health, Faculty of Medicine and Health Sciences, Ghent, Belgium; University of Liège, Division of Diabetes, Nutrition and Metabolic Disorders and Clinical Pharmacology Unit, CHU Liège, Liège, Belgium; Société Scientifique de Médecine Générale (SSMG), Brussels, Belgium; University of Antwerp, Faculty of Medicine and Health Sciences, Primary and Interdisciplinary Care Antwerp (PICA), Antwerp, Belgium

**Keywords:** ■■■

## Abstract

**Background:**

Prescribing that is not concordant with guidelines is increasingly referred to as clinical inertia (CI). However, CI may be only apparent, and the absence of decision may actually reflect appropriate inaction as a result of good clinical reasoning. Our study aimed to: (i) elucidate GPs’ beliefs regarding CI and the risk of CI in their own practice, (ii) identify modifiable provider-related factors associated with CI.

**Methods:**

We conducted 8 group interviews with 114 general practitioners (GP) in Belgium, and used an integrated approach of thematic analysis.

**Results:**

Our results call for a redefinition of CI, in order to take into account the GPs’ extended health-promoting role, and acknowledge that inaction or delayed action follows a process of clinical reasoning that takes into account the patients’ preferences, and that is appropriate most of the time. However, the participants in our study did acknowledge that the risk of CI exists in practice. The main factor of such a risk is when GPs feel overwhelmed and disempowered, due to characteristics of either the patients or the health care system, including contradictions between guidelines and reimbursement policies.

**Conclusions:**

Although situations of clinical inertia exist in practice and need to be prevented or corrected, the term clinical inertia could potentially increase the already existing gap between general practice and specialised care, whereas sustained efforts toward more collaborative work and integrated care are called for.

## Background

Failure to treat to target, or prescribing that is not concordant with guidelines are increasingly being referred to as clinical inertia (CI). Phillips et al. [1] first coined the term, which they defined as a failure to initiate or intensify therapy when indicated, or a failure to act despite recognition of the problem [1]. Alongside patient non-adherence to treatments, CI is believed to be a major factor that contributes to inadequate management of chronic conditions [2,3]. It has been suggested that CI related to management of diabetes, hypertension and lipid disorders may contribute up to 80% of heart attacks and strokes [4]. As it associates with poor control of risk factors known to cause long-term health problems, CI has an economic impact alongside medical consequences [5-7].

O’Connor et al. [2] postulated three classes of factors leading to CI: factors related to (i) providers, (ii) patients, and (iii) the system, with an estimated relative contribution of 50%, 30% and 20% respectively [2]. Other authors report up to 75% provider-related factors [8]. The three provider-related factors that were initially defined by Phillips et al. [1] are assumed to be the most common contributors to CI [2,4,9]: (i) providers’ overestimation of the care they give; (ii) providers’ use of ‘soft’ reasons to avoid therapy; (iii) providers’ lack of education, training or organisation for achieving therapeutic goals.

This list of factors is of little help in overcoming CI in practice. Indeed, practitioners need to be helped to overcome CI rather than systematically blamed for inaction [10], the latter being otherwise occasionally appropriate. Indeed, as summarised by Reach [11], clinical inaction may be called “true” CI only if: (i) a recommendation exists; (ii) the provider knows the recommendation; (iii) the provider believes the recommendation applies to the patient; (iv) the provider has the necessary resources to apply the recommendation; (v) the provider does not apply the recommendation for a particular patient, even though the conditions 1 to 4 are present [11]. Following this definition, non-adherence to guidelines may correspond to appropriate inaction as a result of good clinical reasoning. As shown in our recent literature review [12], actual CI is therefore difficult to observe and distinguish from appropriate inaction. It should not be evidenced without a careful investigation of a practitioner’s reasoning underlying their decisions. Moreover, in order to help anticipate the risk of CI in practice, it is necessary to understand which modifiable and non-modifiable factors underlie CI. Our study aimed to elucidate GPs’ beliefs regarding the risk of CI in their own practice, and personal modifiable factors associated with the risk of CI.

## Methods

We conducted an exploratory qualitative study through group interviews among a sample of general practitioners (GP) in the Wallonie-Bruxelles Region (the French speaking part of Belgium). All participants were members of the same scientific society (*Société Scientifique de Médecine Générale* - SSMG). Over 40% of all GPs in the French speaking part of Belgium are members of SSMG, thus ensuring truthworthiness of our study. As part of their vocational training, members of SSMG from a same geographical area meet regularly around topics of interest to their practice. We used the opportunity of these existing practice-sharing encounters to conduct our interviews. In accordance with the local group coordinators, the GPs were informed beforehand of the topic of the discussion through a letter co-signed by the first author (IA) and the medical coordinator of SSMG. The practitioners who accepted to participate in our study were asked to inform the coordinator of their group that they would participate. We opted for interviews during such formal natural groups [13], rather than purposively sampled groups, as we hypothesized that the GPs would feel more comfortable discussing the issues surrounding CI in the familiar setting of their regular meetings. Our sample involved a total of 114 GPs in 8 group interviews, between October and December 2012. After the 5 first focus groups, a meeting of the steering committee (co-authors) was organised in order to refine the emerging themes and discuss implications for practice. After 3 more focus groups, as no new themes emerged, descriptive saturation of data was reached. The participants’ characteristics are shown in Table 1. Although sampling representativeness may be a less important issue in qualitative research, it is worth stressing that the male/female ratio among the participants in our sample (58.9/41.2) was the same than the male/female ratio among the members of SSMG (59/41).

**Table 1:** **Participants’ characteristics (n = 114)**

	**n(%)**	**Median (range)**
Gender		
Male	67(58,8)	
Female	47(41,2)	
Type of practice		
Single-handed practice	73(64)	
Group practice	34(29,5)	
Mixed (people working in more than one practice)	7(6,5)	
Setting		
Urban	66(58)	
Rural	48(42)	
Work experience		
Median duration of work experience		31,3(2–40)
GPs with less than 10 years of practice	11(9,6)	
GPs with over 35 years of practice	17(15,5)	

### Data collection

A standardised procedure was adopted for the data collection in every group. Two researchers were present at every meeting, and moderated the focus group discussions: The first author would introduce the topic with a brief review of the literature on IC. The group discussion would then be moderated by the second author. The research interviews were guided by two categories of open-ended questions consistent with the objectives of the study: (i) GPs’ beliefs regarding the risk of CI in their own practice, (ii) factors associated with CI. The duration of the discussions was limited to two hours. At the end of every focus group, there was a debriefing between the two researchers to discuss the most important themes that had emerged, and possible similarities and differences to other focus groups.

### Data analysis

All focus groups were audio-taped and transcribed verbatim for analysis. As part of a process of respondent validation and error reduction, every participant received a synthesis of their group’s discussion, and was invited to share comments or further information directly with the researchers, either over the phone or by e-mail. We received feedback from six (6) participants, representing 5 different groups. Three (3) participants thanked the researchers for the discussion and the follow-up, and formally validated the synthesis. Two participants explained how the ideas shared during the focus group were further discussed within their groups, one stressing that it had made him personally more aware of the risk of CI in his own practice. The last participant informed the researchers of a TV programme relevant to the topic of CI. The participants’ comments did not add much to our analysis in terms of thematic contents, but confirmed the relevance of addressing the sensitive issue of CI with GPs.

The transcripts were analysed using an integrated approach of categorisation: A start list of two codes reflecting the research objectives was used as a framework to organise the thematic categories that were inductively created through careful revision of the transcribed material [14,15]. To ensure validity of the findings and interpretations, the first and second authors independently coded the transcripts and met at regular intervals to discuss emerging categories. As already stated, the members of the steering committee (co-authors) were invited to reflect and comment on the analysis process after 5 focus groups. The study was stopped after 8 groups, as no new themes were emerging, meaning that descriptive saturation was achieved. Our results hereafter are illustrated with individual quotations from the various focus-groups [16].

### Ethics

Our study did not involve any patients nor patients’ relatives, nor did it require that patient data be shared with the researchers. Our study does therefore not fall within the scope of the Belgian Law of 7 May 2004 on Human experiments, and did therefore not require the approval of an ethics committee, nor that informed consent forms be signed by the participants.

Ethical considerations were present however at all stages of the project: Every GP was personally informed of the study and invited to knowingly join (or not) the focus group interview several weeks before the encounter; the interview process was conducted with a non-judgmental attitude and was very respectful of the GPs’ perceptions and self-reported experiences regarding the phenomenon of CI in their own practice; last but not least, every GP within a group was sent a synthesis of the discussion within that group, and was thus given the possibility to comment personally, either by e-mail or over the phone, on the synthesis of their interview.

## Results

### GPs’ beliefs regarding CI and the risk of CI in their own practice

To initiate a discussion about CI in general practice raised mixed feelings. It initially made most participants uneasy. In addition to questioning the applicability of guidelines in some situations, the concept of “double-bind” was raised to explain GPs’ difficulty in complying with guidelines in some occasions. Moreover, the GPs’ called for a redefinition of CI, in order to take into account their health-promoting role and to acknowledge that most decisions are taken as a result of a complex process of clinical reasoning, and should not be mistaken for CI.

#### Mixed feelings and perceptions of a double-bind

The GPs generally expressed mixed feelings about the concept of CI, which was new to them. On the one hand they saw the discussion on CI as very interesting, stimulating, and revealing: *“it encourages us to look at our own practice through a critic’s eye” (FG6).* Or: *“Now that I am aware of the risk of CI, will I look differently at my files? Will I change my practice or examine more critically why I don’t change it? I am curious to see what happens…” (FG4).* On the other hand, the topic raised feelings of unease and guilt: “*It’s about what we should do, but currently do not do (…) it’s about feeling guilty for not doing well in some occasions” (FG1).* Quite often the term was even perceived as insulting: *“It’s a very negative term. It conveys that we are passive, that we don’t do anything… (FG4)*”. According to the GPs, the message that is implicitly conveyed with CI is that GPs need to prescribe more. This injunction was perceived as being in total contradiction with the need to comply at the same time with the healthcare system’s demand to reduce costs. The concept of “double-bind” was raised to describe the complexity of the GPs’ role and their feelings of powerlessness in some situations: “*If we strictly prescribed everything as recommended in the guidelines…wouldn’t we be blamed for over-prescribing compared to our colleagues, thus impacting negatively on the budget of the healthcare system?” (FG4).*

#### To care for patients and promote their health, rather than to treat to target

The narrow definition around prescribing to target, was perceived by the GPs as too restrictive in the context of general practice*: “A patient cannot be reduced to figures! Figures alone cannot reflect the complexity of clinical cases. Every situation is unique! We do have targets for our patients, but targets need to be adapted to every patient’s individual situation” (FG2).* The participants believed that a relevant definition of CI needs to include the GPs’ health-promoting role, in addition to that of treating patients. For instance, GPs acknowledged the presence of CI in situations where they might fail to provide timely preventative messages or adequate psychosocial support. They insisted that their role is to care for patients, which is a much broader objective than that of treating to target: “*When my patient is a 75 year-old women with a blood pressure as high as* 150 (mmHg)*, I leave her alone! If she is happy to eat a little piece of cake every day, if she is happy with her life, and her glycaemia is 130 (mg/dL)…well, I won’t bother putting her on medication in order to lower her blood sugar level to 100, and her blood pressure to 13” (FG7).* Guidelines were generally acknowledged as providing state of the art knowledge and were considered very important in indicating a direction rather than presenting a goal to achieve. However, the GPs expressed concern because of a great number of changing and sometimes contradictory guidelines, making it difficult to know what to do in some situations. They also pointed out the fact that highly specialised guidelines may not offer sufficient guidance in cases of co-morbidity. Moreover, the validity of guidelines was questioned by some who thought that new guidelines might be issued in relation with the marketing of new pharmaceutical products.

#### Appropriate inaction versus “true” CI

There was a strong consensus both within the various groups and across the groups that the “failure to initiate or intensify a treatment according to guideline” is a common occurrence in general practice. However, the participants agreed that most decisions are taken after careful examination of the patients’ lives, personal objectives, possibilities and preferences, etc. Moreover, the decision not to prescribe more is justified in situations where the patient is already on a number of different drugs and GPs are aware that the prescription of a new drug might increase the risk of non-adherence or of losing track of a patient. Such decisions should be seen as the result of an appropriate decision following a complex process of clinical reasoning, rather than a manifestation of CI: “*It’s like playing chess: you need to move your pawns step by step to achieve the best results possible instead of charging straight for the king… one move at a time. You mustn’t try to win the game in one go (…) Of course we have targets for our patients, but we adjust them for each individual patient”. (FG2)*

The participants in our study did however acknowledge that the risk of CI exists in practice: “*Some patients do not understand what we mean if we don’t take at least 15 minutes to explain and explain again…We happen to be fed up, and not bother to explain twice.…there we may be blamed for CI”. (FG7)*

### Personal risk factors of CI

Across a variety of situations, a sense of being overwhelmed and feelings of disempowerment were the main common factors associated with the risk of CI.

#### Feeling overwhelmed

The lack of timely investigation to recognise and treat a problem emerged as the most common manifestation of CI, and was reported to be mainly due to factors of human error, such as tiredness, conflicting priorities (private and professional), lack of time, etc. “*At the end of the day, after you saw 20, 30, 35 patients…you had a hard day. If you then see a patient with borderline values, a blood pressure that is not optimal, well, you tend to minimise a bit, you tend to convince yourself that it’s not the right time,that you’ll look at it next time. It’s not an excuse, but nobody is perfect. We also have our children to pick up at school or other conflicting priorities…” (FG5).*

Another reason for not investigating further during a consultation was linked to the attitude of some patients who address multiple demands to the doctor, either for themselves or for other family members: “*Tonight, one of my last patients came because she had flu. She came with her son and husband. At the end of the visit she asked: “Did you receive my son’s and my husband’s blood tests?” By the way, the tests had been done two months ago. The son was OK, but the husband had type 2 diabetes, and his results were not brilliant. I still had one other patient to see before I could go home, prepare supper for my son, and get ready to be on time for our meeting tonight. I told the patient to come back”. (FG8)*

Regarding CI in relation to prescribing medication, an issue frequently reported was that of the amount of administrative work requested for some particular prescriptions: “*You are aware that you should be doing something, but you don’t. Why is that? Because you are tired, because you need to find the right form…I tell you what -as far as I am concerned, my CI is mainly related to paperwork. If I want to prescribe paracetamol, and I need to find the form, which of course is at the bottom of the pile, for the patient to pay 1,50 instead of 3,50…well, sometimes I feel it’s not worth the effort”. (FG7)*

#### Feeling disempowered

A sense of powerlessness was reported to be involved in CI. Patients who are particularly difficult to treat because they are non-adherent, for instance, or aggressive, or denying their medical needs, etc. may induce a sense of powerlessness on the part of the physicians. “*We are there to explain, we take some time with them to explain what their problem is, what diet they should adhere to, what they are exposed to if they don’t change their habits…yet, they do what they want…We do try to increase their motivation regularly. But after some time, some of them make us discouraged and hopeless”. (FG3)*

Moreover, GPs complained about constraints imposed by the healthcare system that sometimes limit their autonomy, and make it difficult to prescribe according to guidelines. A particularly difficult situation for GPs to handle is when they find out that the medication that is recommended according to best practice guidelines is not reimbursed in their country (in Belgium by RIZIV/INAMI, the National Institute for Health and Disability Insurance), or when reimbursement is contingent either on prior authorization from RIZIV/INAMI, or the need that patients visit specialists instead of GPs (as is the case for patients with type 1 diabetes in Belgium).

## Discussion

The results of our group interviews show that non-adherence to guidelines in general practice should not be systematically labeled CI. The Belgian health-care system is characterized by fee-for-service, free access to all levels of care, mostly small single-handed practices in primary care, no compulsory guidelines (which are available but are not strictly “followed”), no real quality assurance, and a tradition of hospital-centered disease-oriented care, all of which can make GPs frustrated about working conditions. Despite this, the GPs in our study displayed great commitment to their work, and believed that prevalent definitions of CI did not take sufficiently into account the active role of the patient in the consultation and medical decision-making process, nor the breadth and complexity of their role, which includes tackling sociocultural determinants of health as much as medical and pharmacological ones.

When situations of CI were actually acknowledged, these were more often connected with a lack of investigation to timely diagnose and treat a problem than with failing to prescribe more. Insufficient delivery of health-promotion and patient-education messages were considered a form of CI as well. Across a variety of cases of CI in which patient and system factors were involved, feelings of being overwhelmed and disempowered was a common issue that emerged as an important factor of CI. Again, appropriate decisions not to adhere to guidelines in specific situations, for specific patients, at specific times are not to be mistaken for CI. As far as prescriptions are concerned, the participants in our study saw their decisions as appropriate most of the time although they were not always concordant with guidelines. Hence, the definition by Philips et al. [1], which emphasizes prescription failures, was considered too narrow, and irrelevant to general practice.

At the heart of evidence-based medicine is the providers’ capacity to integrate individual clinical expertise with the best available clinical evidence from systematic research [17]. Although the concept of CI arises in the context of evidence-based practices, attempts to define it tend to overlook the importance of adjusting care in individual cases. Indeed the main ways of measuring CI, which are based on target, timeframe, and decision to intensify therapy (or not) [2,4] are not sufficient to determine whether individual decisions to increase or not increase therapy might be appropriate for a specific patient [18,19]. As reflected by the participants in our study, in order to adequately assess CI, it is necessary to define *intermediate outcomes* that incorporate information on, and justification of treatment decisions that are made [20]. In the absence of such information, CI may be only apparent, and actually reflect good clinical practice [18].

Application of clinical guidelines in the real world is somewhat problematic because there are practitioner, patient and system variables at play. However, clinicians only have direct control over the first. Finally, the use of the word ‘*inertia*’ to define a concept in Medicine (which would be better described as inaction) may initially have resulted from the mistaken belief that ‘inertia’ in Physics actually implies some obstacle or reluctance in changing motion. Describing the choice to maintain treatment without any change as ‘inert’ can give the incorrect impression of lethargy, and is rather debatable semantically.

The main strengths of our study lie in the number of group interviews and the total number of participants across the country, aiming for saturation of the data and internal validity. Independent coding by two researchers and discussions at several stages with a scientific committee including representatives of several medical specialities and two GPs, are another strength of our study. Regarding the researchers’ characteristics that might have influenced the study, whereas one researcher (first author) is very experienced in conducting qualitative research in the field of health care and clinical communication, the other researcher (second author) is a research nurse with extensive clinical experience and thorough knowledge of evidence-based practice. A strength of the study therefore lies in their complementarity to conduct the study. Moreover, none of them is a medical doctor. Although it might be argued that professions other than physicians are not given enough credence by physicians in other circumstances, we believe that this was a strength for our study, as we approached the physicians without *a priori* representations of how they should be dealing with the risk of CI in their practice. The main limitation lies with our choice of group interviews. Although it enabled us to collect useful information on physicians’ opinions regarding the much debated concept of CI, in-depth individual interviews might have yielded richer data regarding personal experience of CI.

## Conclusions

Our results suggest that non-adherence to clinical guidelines should not be labelled CI in the absence of careful investigation of the patient’s role and the physician’s motives regarding decisions not to act or to postpone therapeutic action in some situations. We believe that the term CI could potentially increase the already existing gap between general practice and specialised care, whereas sustained efforts toward more collaborative work and integrated care are called for.
